# Incidental Appendico-Ileal Fistula in a Patient With Adhesive Intestinal Obstruction: A Case Report

**DOI:** 10.7759/cureus.53369

**Published:** 2024-02-01

**Authors:** Deepak Gusain, Rahul Varshney, Arvind Singh, Jyoti Koli, Rahul Gupta

**Affiliations:** 1 Gastrointestinal Surgery, Synergy Institute of Medical Sciences, Dehradun, IND; 2 Anesthesia and Critical Care, Synergy Institute of Medical Sciences, Dehradun, IND; 3 Gastroenterology, Synergy Institute of Medical Sciences, Dehradun, IND; 4 Internal Medicine, Indresh Hospital, Dehradun, IND

**Keywords:** intraabdominal adhesions, chronic appendicitis, explorative laparotomy, small intestinal obstruction, appendiceal fistula

## Abstract

A fistulous communication between the appendix and any viscus is rare. Such fistula is most often acquired due to recurrent appendicitis, cystic fibrosis, Crohn’s disease, tuberculosis, and malignancy. Here in, we report a rare case of an appendico-ileal fistula incidentally detected during laparotomy for adhesive small bowel obstruction. The fistula was divided, the ileal opening was sutured, and appendectomy was performed. Postoperative recovery was uneventful, with no evidence of malignancy, tuberculosis, or inflammatory bowel disease on the histopathological examination of the appendix.

## Introduction

Acute appendicitis is one of the most common conditions encountered in surgical practice. It can be uncomplicated or complicated [1]. In uncomplicated appendicitis, there is inflammation and edema of the appendix with or without the presence of periappendiceal or pericecal fluid [1]. In complicated appendicitis, there is gangrene of the appendiceal wall with or without perforation and localized or diffuse peritonitis [1,2]. On the other hand, recurrent or chronic appendicitis is an uncommon but well-recognized presentation of appendicitis [3-5]. There is luminal obliteration with chronic inflammatory changes and fibrosis in the appendiceal wall [3,4]. The patient may be asymptomatic or have chronic or recurrent abdominal pain.

Appendiceal fistula is a rare complication of appendicitis. The exact incidence is not known as there are only case reports and series published in the English literature. Depending on the affected organ, fistulous communication has been reported between the appendix and urinary bladder, ileum, rectum, and vagina [6-11]. It can be due to acute or chronic appendicitis, cystic fibrosis, or Crohn’s disease. Preoperative diagnosis of appendiceal fistula is difficult. We report a rare case of an appendico-ileal fistula incidentally detected during surgery for small bowel obstruction.

## Case presentation

A 29-year-old male presented with chief complaints of severe colicky abdominal pain, recurrent bilious vomiting, and constipation for two days. There was no significant past surgical or medical history. The patient denied having tuberculosis in the past. On clinical examination, the patient was hemodynamically stable but dehydrated. The abdomen was distended with sluggish bowel sounds. The patient was resuscitated and a nasogastric tube was inserted for bowel decompression, which had 400 mL of bilious output. Routine blood parameters and urine analysis were within normal limits (Table 1).

**Table 1:** The laboratory parameters of the patient on admission. ALT - alanine transaminase, AST - aspartate aminotransferase, AP - alkaline phosphatase, GGT - gamma-glutamyl transferase

Parameter	Patient’s value	Normal range
Hemoglobin	16.5	13-18 gm/dL
Total leucocyte count	13,680	4000-11,000/cmm
Platelet count	323,000	1.5-4.5 lakh/cmm
Total bilirubin	0.9	0.1-1.2 mg/dL
AST	90	0-40 IU/L
ALT	25	10-50 IU/L
AP	76	40-130 IU/L
GGT	25	8-61 IU/mL
Serum albumin	4.5	3.97-4.94 gm/dL
Blood urea	28	8-50 mg/dL
Creatinine	1.1	0.3-1.3 mg/dL
Sodium	138	136-145 mmol/L
Potassium	4.5	3.5-4.5 mmol/L
Serum calcium	8.7	8.6-10.2 mg/dL
Random blood glucose	93	60-170 mg/dL

**Figure 1:**
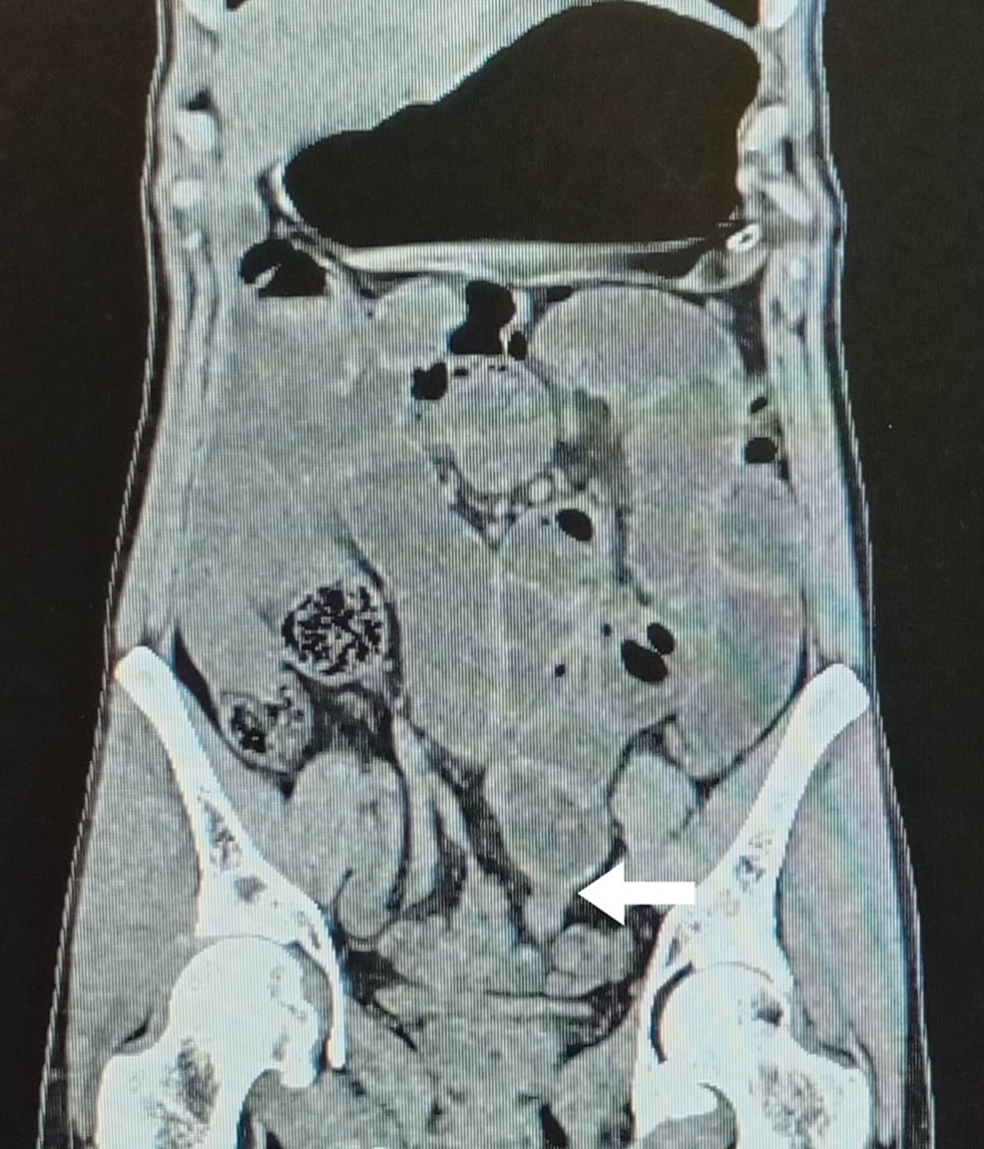
Non-contrast computed tomography of the abdomen showing multiple dilated small bowel loops with the zone of transition in the terminal ileum (white arrow). The appendix could not be identified separately from the ileal loops.

Due to oliguria, non-contrast computed tomography (CT) of the abdomen and pelvis was performed, which revealed multiple dilated jejunal and proximal ileal loops with the zone of transition (Figure 1). The distal ileal and large bowel loops were relatively collapsed. There were multiple enlarged mesenteric lymph nodes measuring 6-14 mm. The appendix could not be visualized.

**Figure 2:**
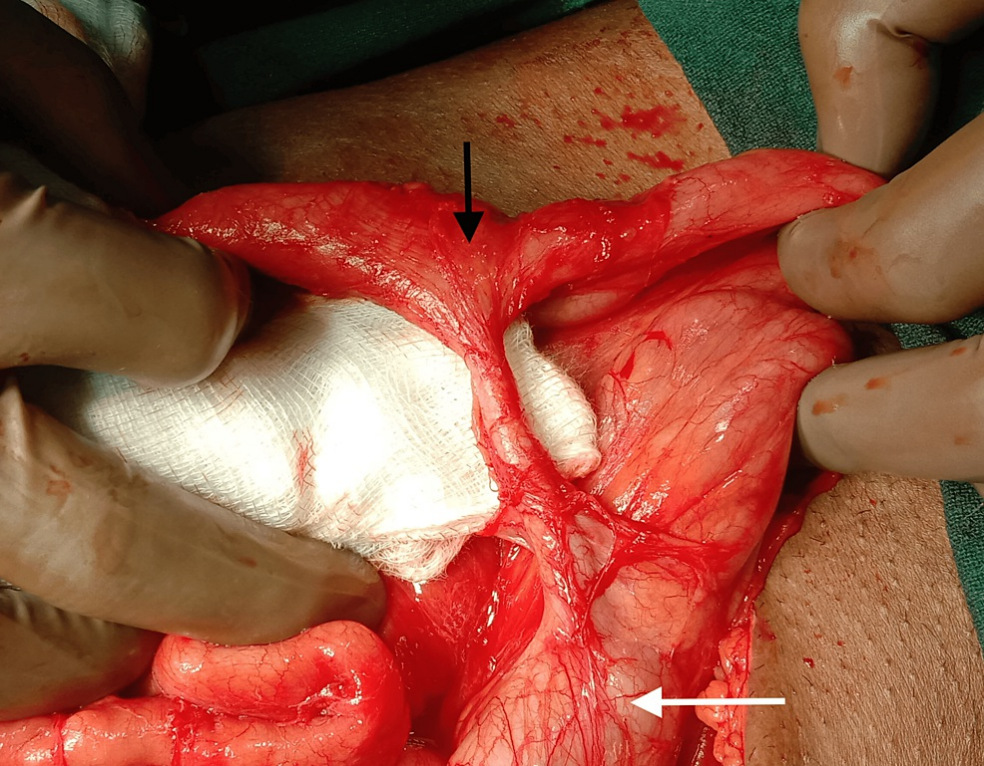
Intraoperative image showing the long appendix arising from the cecum (white arrow) having fistulous communication with the antimesenteric border of the terminal ileum (black arrow).

With the provisional diagnosis of small bowel obstruction, the patient underwent an emergency laparotomy. Intraoperatively, the small bowel loops were found to be densely adhered to each other and the parietal peritoneum causing bowel obstruction in the proximal ileum. Extensive adhesiolysis was performed by sharp and blunt dissection with free passage of the intestinal contents into the terminal ileum. During the adhesiolysis near the ileocecal region, the tip of the appendix was found to be attached to the adjacent ileal loop. On further dissection, an appendico-ileal fistula was identified (Figure 2).

The fistula was divided, the ileal opening was sutured with 3-0 vicryl, and appendectomy was performed. The operative time was 90 minutes with a blood loss of 100 ml. The patient was started on oral liquids from postoperative day two and discharged on a liquid diet on postoperative day four. The histopathology of the appendix showed lymphoid hyperplasia with no evidence of malignancy, tuberculosis, or inflammatory bowel disease. Hence, the exact cause of bowel adhesions and appendico-ileal fistula could not be ascertained. However, previous subclinical appendicular or bowel infections (tubercular or non-tubercular) could have contributed to the development of adhesions and appendico-ileal fistula. On the last follow-up one month after surgery, the patient was doing well on a low-residue semisolid diet. 

## Discussion

Appendiceal fistula is a rare complication of appendicitis. It can occur as a result of acute complicated appendicitis or chronic appendicitis [7,10]. Rarely, it has been reported in patients with cystic fibrosis [6]. The appendix can develop fistulous communication with any intra-abdominal organ, including the colon, rectum, ileum, urinary bladder, ureter, uterus, vagina, etc. [6-11]. Appendico-ileal fistula is very rare with anecdotal case reports in English literature [6,8,9].

The most frequent symptoms are right lower quadrant pain, nausea, vomiting, and pus discharge per rectum or vaginum. In the present case, the fistula was incidentally detected during surgery and was not the cause of intestinal obstruction. The preoperative diagnosis of appendiceal fistula is often difficult. Abdominal ultrasound may reveal a thickened, dilated appendix with the presence of periappendiceal fluid or abscess. Appendicolith may also be seen in some cases. Contrast-enhanced CT abdomen is the investigation of choice for the detection of appendicitis and its complications. It shows the condition of the appendix, its location, appendicolith (if present), surrounding collections, inflammation of the adjacent organs, and, rarely, the fistulous tract [7,8,10]. Alternatively, an MRI abdomen can also be used to detect appendiceal fistula [10]. Colonoscopy is a useful investigation to detect colonic pathologies, visualization of the appendiceal orifice, and the opening of the fistulous tract in case of appendico-colonic or appendico-rectal fistula [10]. Cystoscopy helps identify the site of an appendico-vesical fistula [11].

Surgery remains the mainstay of treatment for appendiceal fistula. The crucial steps of the surgery include the dismantling of the fistula, appendectomy, and primary repair of the affected organs, such as the ileum, vagina, or bladder. The surgery can be performed by minimally invasive techniques [11], but in most of the previous reports, open surgery was performed due to its complexity [10]. Alternatively, percutaneous drainage can be offered to patients with appendicular abscesses who are hemodynamically unstable or not suitable for emergency surgery [12]. Percutaneous drainage will help control the sepsis and allow time to plan the surgery. The surgery can be delayed up to six weeks in such cases.

## Conclusions

Appendico-ileal fistula is a rare condition. It is very difficult to diagnose preoperatively because of non-specific symptoms and inconsistent radiological findings. Appendix must be examined whenever feasible in patients undergoing abdominal surgery to rule out appendiceal fistula. Dismantling of the fistula and appendectomy must be performed to prevent future complications.
